# Resilience as a predictor of quality of life in participants with borderline personality disorder before and after treatment

**DOI:** 10.1186/s12888-021-03312-0

**Published:** 2021-06-12

**Authors:** Verónica Guillén, Mireia Esplugues Tormo, Sara Fonseca-Baeza, Cristina Botella, Rosa Baños, Azucena García-Palacios, José Heliodoro Marco

**Affiliations:** 1 Department of Personality, Evaluation and Psychological Treatment, University of Valencia, Av. Blasco Ibañez 21, 46010 Valencia, Spain; 2 CIBER of Physiopathology of Obesity and Nutrition (CB06/03) Instituto Salud Carlos III, Madrid, Spain; 3 Department of Basic and Clinical Psychology and Psychobiology, Jaume I University of Castellon, Castellon, Spain

**Keywords:** Personality disorder, Psychological treatment, Dialectical behavior therapy, Systems training for emotional predictability and problem solving, Resilience, Quality of life

## Abstract

**Background:**

Studies have suggested that psychotherapy improves the Quality of Life (QoL) of participants with Borderline Personality Disorder (BPD). However, there are no studies on the differential efficacy of treatments on the QoL of participants with BPD. Moreover, the relationship between QoL and resilience has rarely been studied in participants with BPD. Objectives: a) to examine whether people with BPD have worse QoL than the non-clinical population; b) to examine whether there are statistically significant differences between Dialectical Behavioural Therapy (DBT), Systems Training for Emotional Predictability and Problem Solving (STEPPS), or Cognitive Behavioural Therapy-Treatment at Usual (CBT-TAU) in the improvement of QoL; c) to examine whether participants show clinically significant improvements in QoL after treatment; d) to analyse whether resilience is associated with QoL before and after the BPD treatment; e) to analyse whether resilience is a predictor of QoL at pre-treatment and posttreatment.

**Method:**

The sample comprised 403 participants (*n* = 202 participants diagnosed with BPD and *n* = 201 non-clinical). Participants filled out the Quality of Life Index, Resilience Scale, and Beck Depression Inventory. The clinical participants received one of these possible treatments, DBT, STEPPS, or CBT-TAU. MANOVA and regression analyses were performed.

**Results:**

a) participants diagnosed with BPD had statistically significant lower resilience than the non-clinical population; b) all three forms of psychotherapy statistically improved QoL, but there were no statistically significant differences between DBT, STEPPS, and CBT-TAU in the improvement of QoL; c) participants did not show clinically significant improvements in QoL after treatment; d) resilience was associated with QoL before and after treatment; and e) resilience was a predictor of QoL before and after treatment.

**Conclusion:**

It is necessary to assess QoL and Resilience in studies on psychotherapy with BPD patients.

## Background

There is a broad consensus in the research that Quality of life (QoL) is a multidimensional construct that can be defined as “an overall general well-being that comprises objective descriptors and subjective evaluations of physical, material, social, and emotional wellbeing together with the extent of personal development and purposeful activity, all weighted by a personal set of values” [1].

Borderline personality disorder (BPD) is characterized by instability in interpersonal relationships, self-image, affects, and impulse control, severe functional impairment, and a high risk of suicide [2]. Moreover, BPD is associated with high comorbidity with other mental disorders, such as eating disorders, post-traumatic stress disorders, mood disorders, anxiety disorders, and other personality disorders [3, 4].

Perseius, Andersson, Asberg and Samuelsson [5] found that QoL scores in Swedish participants with BPD were more than one standard deviation below the scores of the non-clinical population. Lawford and Eiser [6] suggest that when patients rate their QoL, they place greater emphasis on mental functioning than on physical functioning. Therefore, BPD clinical features could affect and worsen QoL in individuals with a BPD diagnosis. Indeed, Cramer, Torgersen and Kringlen [7] found that people with BPD (along with avoidant, schizotypal, schizoid, and paranoid personality disorders) had poorer QoL compared to those with no BPD, and low QoL was associated with lower subjective well-being and more negative life events. Other studies found that QoL declines due to: the symptomatology of the disorder, comorbidities with other mental conditions [8], suicide attempts and self-harm [9], hospitalizations [10], physical illnesses [11], shame, low self-esteem, anger, and hostility [12]. However, Thompson et al., [13] in a recent study with young participants with BPD, found that depression symptoms were the best predictor of worse QoL, and that frequency of hospitalizations, suicide attempts, and non-suicidal self-injuries (NSSI) were not associated with QoL. Despite results suggesting that QoL is highly impaired in people with BPD, the research on QoL in BPD is scarce.

QoL is an important indicator of the outcome of treatment interventions in several mental disorders, such as bipolar [14] or panic disorders [15]. However, QoL is rarely assessed as an outcome measure in efficacy treatment studies of BPD [16]. Treatment effectiveness in BPD has usually been measured as a change in the symptomatology (i.e. improvement or reduction), such as NSSI, suicide attempts and frequency of hospitalizations, and social functioning. Nonetheless, many patients’ needs are not met [17], and symptom reduction does not always translate into restoration of QoL to normal levels.

In the case of Dialectical Behavioural Therapy (DBT), only two randomized controlled trials (RCTs) have analysed the effectiveness of DBT for QoL, finding that QoL improved after DBT. McMain et al. [18] found that DBT had similar effects on QoL improvement as general psychiatric management, and Carter et al. [19] found that DBT more significantly improved QoL, compared to treatment as usual (TAU). In addition, Systems training for emotional predictability and problem solving (STEPPS) was found to be more effective than TAU in improving QoL [20]. In the case of Cognitive-behavioural therapy (CBT), three RCTs analysed the effects of CBT on QoL and found that CBT groups had sustained QoL improvement, but not significantly different from TAU [21] or Rogerian supportive therapy [22]. Finally, two RCTs compared Schema-focused therapy to Transference-focused therapy [23], and both groups improved their QoL, with differences between the two conditions depending on the QoL outcome measure [24, 25]. Nadort et al. [26] found that Schema-Focused Therapy was effective in improving QoL, independently of telephone therapist availability during crises. Finally [27], a meta-analysis of the efficacy of BPD psychotherapy on QoL found that psychotherapy produced improvements in QoL, with a moderate effect size of d = 0.32 (95% CI [0.17, 0.48]. Thus, the aforementioned studies suggest that all the forms of psychotherapy improved QoL, but none of the treatment studies examined whether the improvements in QoL represented clinically significant changes [28] in the participants’ QoL [16]. To find out whether the treatment resulted in clinically significant change, two conditions are necessary: first, analyse whether reliable change was achieved; and second, compare the difference between post-treatment clinical scores and the scores of healthy populations on the measures utilized. The change can only be considered clinically significant when the treatment brings about a reliable change and the outcomes become similar to those found in healthy populations [28].

Resilience can generally be defined as ‘the capacity of a dynamic system to withstand or recover from significant challenges that threaten its stability, viability, or development’ [29], and it is a dynamic process that leads to successful individual adjustment in the face of adversity [30]. The relationship between QoL and resilience has been widely studied in chronic disease [6], the human immunodeficiency virus [31], multiple sclerosis, [32], and cancer [33]. In mental disorders, several studies found that resilience was a significant predictor of QoL in individuals with schizophrenia, bipolar disorder, and healthy controls, such that higher resilience led to higher QoL [34–36].

Resilience has rarely been studied in participants with BPD (e.g., [37]). Several authors [38] suggest that the absence of resilience is a core characteristic of BPD, and it results from inflexibility in the human capacity for social communication and difficulties with reappraisal when facing negative experiences in social interactions. Thus, all the effective treatments are effective because they open up the patient to social learning experiences, and therapeutic change is probably due to the way patients come to use their social environment. One consequence of this theory is that effective treatment would improve the resilience of participants with BPD and be associated with a reduction in patients’ symptoms and an increase in QoL.

Nevertheless, as far as we know, there are no studies that explore the association between resilience and QoL in participants with BPD. Thus, the objectives of the study are: a) to examine whether people with BPD have worse QoL than the non-clinical population; b) to examine whether there are statistically significant differences between DBT, STEPPS, and TAU-CBT in the improvement in QoL; c) to examine whether participants show clinically significant improvements in QoL after treatment; d) to analyse whether resilience is associated with QoL before and after the BPD treatment; and e) to analyse whether resilience is a predictor of QoL at pre-treatment and posttreatment.

Based on previous studies, we hypothesize that: a) people with BPD will have worse QoL than the non-clinical population; b) all the types of psychotherapy will improve QoL; c) after treatment, participants will not show clinically significant improvements in QoL; d) resilience will be strongly associated with QoL before and after the BPD treatment; e) resilience will be a predictor of QoL before and after the BPD treatment.

## Method

### Participants

The clinical sample comprised 202 Spanish participants diagnosed with BPD, 80.7%, *n* = 163, of whom were female. The mean age was 28.93 years (*SD* = 9.40). Regarding their marital status, 46.5%, *n* = 94, were married or had a partner, and 53.46%, *n* = 108, were single, divorced, or widowed. Regarding the educational level, 2.5%, *n* = 5, had no studies; 27.7%, *n* = 56, had primary school level studies; 48%, *n* = 97, had a high school education; and 21.8%, *n* = 44, had university level studies. Regarding the participants’ psychiatric comorbidity, 78.2%, *n* = 158, matched the criteria for another mental disorder. Of them, 62.5%, *n* = 127, met eating disorder criteria; 6.5%, *n* = 13, met abuse substance disorder criteria; 3.6%, *n* = 7, met obsessive compulsive disorder criteria; 2.9%, *n* = 6, met anxiety disorder criteria; and 2.7%, *n* = 5, met mood disorder criteria. The mean score on the Global assessment of functioning from the DSM-IV was 56.18 (*SD =* 33.69). The number of inpatient hospitalizations in the past year was 0.84 (*SD =* 1.87), the frequency of suicide attempts in the past year was 0.41 (*SD =* 0.98), and the frequency of non-suicidal self-injuries (NSSI) in the past year was 2.89 (*SD =* 7.63). Moreover, 34.2% presented a physical illness.

The inclusion criterion was: patients who met the full DSM-IV [39] criteria for BPD. The exclusion criteria included moderate or severe intellectual disability and meeting the criteria for schizophrenia or another psychotic disorder. Participants were European Whites. They were recruited consecutively. Participants were volunteers who did not receive any compensation for their participation, and they signed an informed consent form. Ethical approval to carry out this study was granted by the University Ethics Committee of Clinical Studies.

The non-clinical sample comprised 201 Spanish university students without mental disorder diagnoses; 72.6%, *n* = 146, were female. Regarding their marital status, 38.3%, *n* = 71, were married or had a partner, and 61.7%, *n* = 124, were single. Participants ranged in age from 18 to 60 years, with a mean age of 22.37 years (*SD* = 5.42). Participation was voluntary, and they did not receive any compensation. Informed consent was obtained from all participants.

### Instruments

Structured clinical interview for DSM-IV axis I disorders (SCID I) [40]. This is an interview for making the major DSM-IV-TR [39] Axis I diagnoses. It offers good psychometric properties: Kappa .66, demonstrating reliability [41]. The Spanish version shows psychometric properties similar to those of the original scale [42].

Structured clinical interview for DSM-IV axis II personality disorders (SCID II) [43]. This is an interview for making DSM-IV-TR [39] Axis II Personality Disorder diagnoses. It includes 119 questions and has a Kappa of .74, demonstrating reliability for admitted patients [43]. The Spanish version shows psychometric properties similar to those of the original scale [44].

#### Relevant clinical information inventory

Created ad hoc for this research, it collects the frequency of NSSI (from 0 to the maximum number of NSSI). NSSI were conceptualized as self-injurious behaviours that were not intended to be an attempt to end one’s life. The number of NSSI in the year prior to the initial assessment was assessed through an open question: Have you ever caused yourself any self-directed and deliberate injuries, such as cutting, hitting, scratching etc., with no suicidal intent? (yes/no). How many times/days have you caused yourself such injuries in the past year? Suicidal Attempts were conceptualized as self-inflicted, potentially injurious behaviours with a non-fatal outcome, but with evidence of the intention to die [45]. The number of suicide behaviours in the past year was assessed with the following question, created ad hoc for this research: Have you ever tried to end your life? (yes/no). How many times have you attempted suicide in the past year? The responses related to methods were categorized by clinical psychologists. Moreover, we collected the frequency of inpatient hospitalizations in the past year.

Quality of life index (QLI) [46]. QoL was assessed by the Spanish version [47] of the QLI, which consists of 10 items that can be rated from 0 (poor) to 10 (excellent). Each item represents one relevant dimension of QoL: Physical Well-being, Psychological/Emotional Well-being, Self-care and Independent Functioning, Occupational Functioning, Interpersonal Functioning, Social Emotional Support, Community and Services Support, Personal Fulfilment, Spiritual Fulfilment, and Overall Quality of Life. Each dimension contains a brief explanation in parentheses, designed to allow flexibility in the interpretation depending on the individual’s cultural and experiential background [48]. The final score ranges from 1 to 10 and is obtained by calculating the average of the scores on all the items. The translation of this scale has shown satisfactory test-retest reliability (Cronbach’s α = 0.89) and validity properties, and in our sample, the reliability was adequate (α = 0.88).

Resilience Scale (RS-15) [49] Resilience was measured by the short form of the Spanish version of the Resilience Scale [50], which was originally developed by Wagnild and Young [51]. The Spanish version replicated the bi-factorial structure of the original scale (“personal competence” and “acceptance of self and life”). Cronbach’s α for the total scale was .93 for the general population and .94 for patients with eating disorders. The RS-15 is a 15-item self-report measure of resilience, defined by the authors as a positive personality trait that promotes adaptability amid adversity. Each item is rated by the participant using a 7-point Likert scale ranging from disagree [1] to agree [7]. Possible scores range from 15 to 105, with higher scores indicating higher perceived resilience. Psychometric properties and internal consistency are adequate and similar to the original scale [49]. In our sample, the reliability was adequate (α = 0.93).

#### Beck depression inventory-II (BDI-II) [52]

This inventory consists of 21 items with four response options (0–4) that rate depressive symptomatology. Its Spanish version offers good psychometric properties [53]. It has presented adequate reliability (Cronbach’s α = 0.90) in Spanish participants. In our sample, the reliability was adequate (α = 0.91).

### Procedure

The clinical sample was collected from treatment-seeking patients in three Specialized services in personality disorders in Spain between 2011 and 2018. All participants were informed about the study and gave their written informed consent. Several expert clinical psychologists with more than 10 years of experience with BPD treatment conducted the assessment to ensure that patients met the inclusion criteria. The participants received one of these possible treatments, depending on their clinical situation and the study underway in the clinical centres at the time of recruitment: DBT [54], STEPPS [55], or TAU-CBT. DBT is a treatment with broad empirical support for BPD [56]. It draws on dialectical tensions of the behaviours, which can be functional and dysfunctional at the same time, and it targets a balance between acceptance and change [57]. It consists of a combination of individual psychotherapy, group skills training, telephone coaching, and a therapist consultation team [58]. STEPPS is a cognitive behavioural model in group format that incorporates skills training and creates a common language for the patients and their system [55]. In this treatment, maladaptive schemas are confronted, and BPD is reframed as an emotional intensity disorder [59]. TAU-CBT is the standard treatment protocol in clinical centres; namely, it is a cognitive behavioural program focused on treating the symptomatology by using CBT strategies. In our study, it consisted of one hour of individual therapy – in which personality psychopathology symptoms were also addressed – and one two-hour group session per week. The TAU-CBT group format was adapted by the clinical team and included psychoeducation, cognitive restructuring, and consolidation of achievements. All these programs lasted about 6 months, and patients completed self-reports on resilience and QoL both before and after the treatment. All the psychotherapeutic groups received a similar number of psychotherapy sessions.

For the nonclinical sample, a convenience sample of university students was recruited. The samples were matched on age. They completed the questionnaires during their normal day at the university.

### Data analysis

Means and standard deviations and zero-order correlations were calculated for all the variables at baseline (T1) and at post-treatment (T2). First, to calculate whether there were differences in QoL between the non-clinical population and the participants with a diagnosis of BPD, a t-test was performed. Second, to examine whether there were statistically significant differences between DBT, STEPPS, and TAU in the improvement in QoL after treatment, a MANOVA was performed, and the effect sizes were calculated (Cohen’s d). Moreover, we analysed whether the treatments produced a clinically significant change in QoL Three conditions are necessary to consider a change clinically significant [28]: a) the Reliable change index (RCI) is calculated; RCI is a methodology that indicates whether the change detected after a treatment represents a real modification in the patient’s clinical condition, or if it simply reflects a measurement error surrounding test-retest difference scores. If there is a reliable change, the assumption can be made that changes are due to treatment and not to a measurement error; b) after treatment, the QoL scores should be situated in the mean range of the normal population (+/− SD) to interpret the functional direction [60]; and c) the effect size is calculated using Hedge’s *g* with the range of a normal population to confirm that there are no statistically significant differences in QoL after the treatment [61].

Finally, we performed two linear regression analyses. In the first model, we took Resilience before treatment (RS T1) as the predictor variable and QoL pre-treatment (QoL T1) as the dependent variable. In the second linear regression analysis, we took Resilience after treatment (RS T2) as the predictor variable and QoL post-treatment (QoL T2) as the dependent variable. In the two prediction models, Type of psychotherapy, Gender, Age, and depression (BDI-II) were controlled. Potential multicollinearity between prediction variables was rejected due to tolerance values and a variance inflation factor between 0.9 and 1.3, respectively, which meet good statistical criteria [62]. Data were analysed using SPSS 24 [63].

## Results

### Participants’ flow

**Fig. 1:**
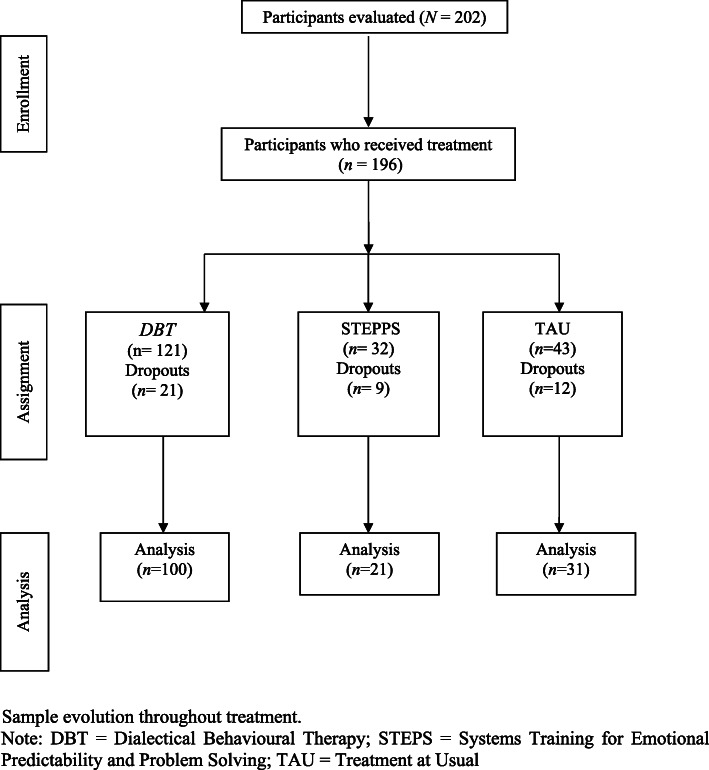
Sample evolution throughout treatment. DBT = Dialectical Behavioural Therapy; STEPPS = Systems Training for Emotional Predictability and Problem Solving; TAU = Treatment as Usual

Figure 1 displays the participant flow diagram during treatment. There were 202 participants assessed at pre-treatment. In the initial sessions, six participants who chose not to attend the therapy groups were excluded from further treatment. Finally, 196 participants received psychotherapy: *n* = 121 received DBT, *n* = 32 received STEPPS, and *n* = 47 received TAU-CBT. During the treatment, in the DBT group, 17.35% (*n* = 21) dropped out, in the STEPPS group, 28.12% *(n* = 12) dropped out, and in the TAU-CBT group, 27.91% (*n* = 12) dropped out. Thus, the percentage of overall treatment dropout was 23.46% (*n* = 46). The clinical sample was older than the non-clinical sample (*t*_(391)_ = 8.26, *p* < .001) (Cohen’s *d* = 0.9).

### Differences in QoL between the clinical and non-clinical samples

Before beginning treatment, participants diagnosed with BPD had a statistically significant lower QoL (QoL T1) (*M* = 4.31, *SD* = 1.74) than the non-clinical population (*M* = 7.86, *SD* = 1.24) (t_(352.44)_ = 23.34, *p* < .001), with a large effect size (Cohen’s *d* = 2.37) [64]. In the same way, participants diagnosed with BPD had statistically significant lower resilience (RS T1) (*M* = 51.76, *SD* = 18.93) than the non-clinical population (*M* = 86.61, *SD* = 11.19) (t_(267.15)_ = 21.10, *p* < .001), with a large effect size (Cohen’s *d* = 2.24).

### Change during psychotherapy

As Table 1 shows, after the treatment, all the participants significantly increased their QoL scores (*F*_(1,144)_ = 11.81, *p* < .001). However, there were no statistically significant differences between DBT, STEPPS, and TAU in the improvement in QoL after treatment (*F*_(1,144)_ = 0.31, *p* = .73). Moreover, participants did not show clinically significant improvements in QoL at post-treatment because no reliable changes occurred (Reliable change index = 1.53, *p* > .05), and the scores were not similar to those of the non-clinical population (range 6.62 to 9.1), with a moderate effect size (Hedges’ *g* = 0.39). Thus, the participants diagnosed with BPD still had lower QoL than the non-clinical sample (*t*_(239,06),_ *p* < .001) after the treatment.

**Table 1:** Pre-treatment and post-treatment means and standard deviations

Variable	Pre-treatment		Post-treatment		Pre-post-Treatment change				
	M	SD	M	SD	M	SD	t (118)	*p*	*Cohen’s d*
QoL	4.31	1.74	4.98	1.97	−0.66	19.85	−4.01	.001	0.35
Resilience	51.76	18.83	58.92	20.76	−7.92	2.02	−4.35	.001	0.39
Depression	32.75	15.17	26.17	16.79	6.58	15.75	5.08	.001	0.41

Note. QoL = Quality of Life

After the treatment, the participants significantly increased their resilience (t_(118)_ = − 4.35; p < .001) and significantly decreased their depression (t_(118)_ = 5.08; p < .001). As Table 1 reveals, all the effect sizes were moderate (range Cohen’s *d* = 0.35–0.41).

As Table 2 shows, resilience at pre-treatment (RS T1) was highly and positively correlated with QoL (QoL T1), and highly and negatively correlated with depression (BDI-II T1) and resilience at post-treatment (RS T2). Moreover, resilience at pre-treatment (RS T1) was moderately and positively correlated with QoL post-treatment (QoL T2) and moderately and negatively correlated with depression post-treatment (BDI-II T2). Resilience at post-treatment (RS T2) was highly and positively correlated with QoL (QoL T2) and highly and negatively correlated with depression (BDI-II T2). Table 2 presents the rest of the correlations.

**Table 2:** Zero-order correlations between the variables

	2	3	4	5	6
1Resilience T1	.74**	−.66**	.50**	.38**	−.45**
2 QoL T1		−.67**	.42**	.41**	−.42**
3 BDI-II T1			−.43**	−.28**	.52**
4 Resilience T2				.74**	−.66**
5 QoL T2					−.60**
6 BDI-II T2					

Note: QoL = Quality of Life; BDI-II = Beck Depression Inventory II; T1 = pre-treatment assessment; T2 = post-treatment assessment

As Table 3 shows, the model composed of resilience before the treatment (RS T1), Type of psychotherapy, Gender, Age, and depression (BDI-II T1) predicted QoL pre-treatment (T1) (*R*^*2*^ adjusted = .64; *F*_(5.153)_ = 71.94, *p* < .001). After entering Type of psychotherapy, Gender, Age, and depression (BDI-II T1), Resilience before the treatment (RS T1) predicted QoL pre-treatment (T1) (*ΔR*^2^ = .16). As Table 3 shows, when analysing the individual contribution of each predictor variable, the variables that significantly predicted QoL (T1) were Resilience pre-treatment (RS T1) (*t* = 8.48; *p* = .01) and depression pre-treatment (BDI-II T1) (*t* = − 5.26; *p* = .01).

**Table 3:** Hierarchical regression analyses predicting QoL before the treatment

Step	Variable entered	Β	Standard error	t	Total R^2^	ΔR^2^
1	Type of Psychotherapy	−0.31	.17	−1.77		
	Gender	0.10	.33	0.31		
	Age	−0.01	.01	−1.54		
	BDI T1	−0.04**	.01	−5.25	.48**	.16**
2	RS T1	0.05**	.01	8.48	.64**	

Note. QoL = Quality of Life; BDI = Beck Depression Inventory; RS = Resilience Scale; T1 = Before psychotherapy. *p < .01, **p < .001

As Table 4 shows, the model composed of Resilience after the treatment (RS T2), Type of psychotherapy, Gender, Age, and depression (BDI-II T2) predicted QoL post-treatment (T2) (*R*^*2*^ adjusted = .58; *F*_(5.118)_ = 59.89, *p* < .001). After entering Type of psychotherapy, Gender, Age, and depression (BDI-II T2), Resilience after the treatment (RS T2) predicted QoL posttreatment (T2) (*ΔR*^2^ = .21). As Table 4 shows, when analysing the individual contribution of each predictor variable, the variables that significantly predicted QoL (T2) were Resilience posttreatment (RS T2) (*t* = 7.73; *p* = .001) and depression post-treatment (BDI-II T2) (*t* = − 2.47; *p* = .01).

**Table 4:** Hierarchical regression analyses predicting QoL after the treatment (QoL T2)

Step	Variable entered	Β	Standard error	t	Total R^2^	ΔR^2^
1	Type of Psychotherapy	−0.04	.24	−.19		
	Gender	−0.23	.49	−.48		
	Age	0.01	.01	.16		
	BDI T2	−0.02*	.01	−2.47	.37**	.21**
2	RS T2	0.05**	.01	7.38	.58**	

Note. QoL = Quality of Life; BDI = Beck Depression Inventory; RS = Resilience Scale; T1 = Before psychotherapy. *p < .01, **p < .001

## Discussion

The objectives of the study were: a) to examine whether people with BPD had worse QoL than the non-clinical population; b) to examine whether there were statistically significant differences between DBT, STEPPS, and TAU in the improvement of QoL; c) to examine whether participants showed clinically significant improvements in QoL after treatment, and whether the scores were within the range of the non-clinical population; d) to analyse whether resilience was associated with QoL before and after the treatment for BPD; and e) to analyse whether resilience was a predictor of the QoL at pre-treatment and posttreatment.

Regarding the first aim, we found that participants diagnosed with BPD had lower QoL than the non-clinical population. Spanish participants with BPD were more than one standard deviation below the non-clinical population. This result is consistent with several previous studies [5, 7, 12, 65, 66] that found that participants with BPD had low QoL. Moreover, participants diagnosed with BPD had lower resilience than the non-clinical population. Spanish participants with BPD were more than one standard deviation below the non-clinical population.

Regarding the second aim, our results indicated that all three forms of psychotherapy (DBT, STEPPS, and TAU-CBT) improved QoL. These results are congruent with a previous review study on QoL [16]. However, in our study, we compared the effects of three different types of psychotherapy on improving QoL, and we found that there were no differences between the different psychotherapies.

Regarding the third aim, our results suggest that, although the improvement in QoL after treatment was statistically significant, it was certainly small, and the treatments did not raise it to normal levels; thus, there was no clinical change in QoL after the treatments. This result points out how critical it is to intervene and allocate resources to improving the QoL of BPD patients. Even though treatments improve their QoL, the current treatments for BPD are not effective in making a clinical change in QoL (see [16]).

Regarding the fourth aim, our results support the hypothesis that higher resilience is associated with higher QoL. A positive relationship was found between resilience and QoL in BPD patients before and after the psychotherapy. Finally, resilience was a predictor of QoL before and after the treatment, controlling for the type of psychotherapy, gender, age, and the change in depression.

Our results are consistent with previous research on the relationship between QoL and resilience in other disorders. For instance, Rainone et al. [32] investigated the role of resilience in improving the QoL of young adults with multiple sclerosis. They found that their measure of resilience as a process moderated the relationship between depression and QoL. The introduction of resilience in their model decreased the negative effect of depression on affective functioning. The positive relationship between resilience and QoL has also been reported in other mental disorders [34, 36], and resilience has already emerged as a predictor of QoL [35].

Our study could support the theory of Fonagy et al. [38], who suggest that the absence of resilience is a core characteristic of BPD, and that therapy helps because resilience improves during treatment. This change in resilience has an impact on QoL as well, which suggests that by targeting the improvement in resilience in BPD patients, clinical psychologists will help them to have better QoL. This reaffirms our conceptualization of resilience as a dynamic process – sensitive to change – and should encourage the scientific community to begin to more thoroughly investigate the relationship between resilience and QoL in BPD patients. It would be interesting to study which specific components of resilience influence each dimension of QoL because both constructs are multidimensional.

Our study has some limitations. First, different operational definitions of QoL and resilience make it harder to compare our study with other studies. There is a need for unification, which many authors have tried (e.g. [67]), but there is still no consensus among the scientific community. Second, the sample sizes in the different treatment conditions were not similar, and so it is possible that with larger sample sizes that are similar across the conditions, we could find statistically significant differences between treatment conditions. Thus, future studies should replicate our study with larger and more similar sample sizes between conditions. Moreover, the non-clinical sample was significantly younger, and the percentage of women was higher than in the non-clinical sample. Thus, future research should compare BPD samples with non-clinical samples matched on age and gender to confirm our results. Third, our study is not a randomized controlled trial, and so we only can suggest associative relationships between the variables analysed, and never causal relationships. Fourth, in our sample, 62.5% of the participants had a comorbid diagnosis of an eating disorder. Therefore, these results would only be generalizable to samples of participants with BPD and comorbid ED. Moreover, we did not assess the severity of BPD with a validated instrument, and it is very important to assess BPD severity because it may indicate both resilience and quality of life. Finally, another limitation is that we did not use a scale to assess NSSI, and we cannot confirm the reliability of this measure in our sample. However, a review study [68] found that the type of NSSI assessment, whether with a single item or with self-report questionnaires, did not influence the assessment. Our results suggest that resilience plays a key role in predicting the QoL of BPD patients. Thus, it is recommended to further explore resilience in the BPD community, especially in treatment contexts, and investigate the impact of these interventions in the long term. In this regard, three primary questions would need attention: 1) What strategies improve resilience skills?; 2) How does implementing resilience skills in therapy influence treatment effectiveness?; and 3) Are these results clinically relevant as well as statistically relevant? As far as we know, this is the first study to explore the relationship between resilience and QoL in BPD patients. Our study is just preliminary. It would be interesting to see if future research replicates our results. In addition, taking into account that the concept of QoL is quite broad, future research should investigate subdomains of resilience and their impact on the different dimensions of QoL.

## Conclusions

In summary, our study suggests that it is necessary to evaluate QoL in studies on treatments for people with BPD. Future research should also analyse the reasons for this slight improvement in QoL after treatment. These results indicate that resilience plays an important role as a predictor of QoL in BPD patients. Because both resilience and QoL tend to be low, working on resilience and QoL should be two therapeutic targets of intervention programs. Future studies should address more specific questions and expand our knowledge about resilience and QoL in participants with BPD.

## Data Availability

The datasets used and/or analysed during the current study were collected as part of a doctoral thesis and are available from the corresponding author on reasonable request.

## Abbreviations

BPD: Borderline Personality Disorder
CBT: Cognitive-Behavioural Therapy
DBT: Dialectical Behavioural Therapy
BDI-II: Beck Depression Inventory II
NSSI: Non-suicidal self-injuries
QLI: Quality of Life Index
QoL: Quality of Life
RCTs: Randomized controlled trials
RS: Resilience Scale
SCID I: Structured Clinical Interview for DSM-IV Axis I Disorders
SCID II: Structured Clinical Interview for DSM-IV Axis II Personality Disorders
STEPPS: Systems Training for Emotional Predictability and Problem Solving
TAU: Treatment as usual
